# Does wearing arthritis gloves help with hand pain and function? A qualitative study into patients’ views and experiences

**DOI:** 10.1093/rap/rkac007

**Published:** 2022-02-12

**Authors:** Yeliz Prior, Carol Bartley, Jo Adams, Jill Firth, June Culley, Terence W O’Neill, Alison Hammond

**Affiliations:** 1 Centre for Health Sciences Research, School of Health and Society, University of Salford, Salford; 2 Rheumatology, Mid Cheshire NHS Hospitals Trust, Leighton Hospital, Crewe; 3 Rehab for Independence Ltd, Heskin; 4 School of Health Sciences, University of Southampton, Southampton; 5 Pennine Musculoskeletal Partnership, Oldham; 6 Patient Research Partner, Derby; 7 Centre for Epidemiology Versus Arthritis, University of Manchester; 8 Manchester Academic Health Sciences Centre, NIHR Manchester Biomedical Research Centre, Manchester University NHS Foundation Trust, Manchester, UK

**Keywords:** arthritis gloves, compression gloves, RA, inflammatory arthritis, hand pain, hand problems, functional limitations, orthoses, occupational therapy

## Abstract

**Objective:**

Arthritis gloves are frequently prescribed to people with undifferentiated inflammatory arthritis (UIA) or RA to help reduce hand pain and improve function. Nested within a randomized controlled trial testing the effectiveness of arthritis gloves (Isotoner gloves *vs* loose-fitting placebo gloves) in people with RA and UIA, this qualitative study aimed to explore participants’ views on the impact of wearing arthritis gloves on their hand pain and function.

**Methods:**

Semi-structured one-to-one interviews were conducted with purposively selected participants following 12 weeks of glove wearing. Participants and the interviewer were blinded to the treatment allocation. Interviews were audio-recorded, transcribed verbatim and analysed using thematic analysis.

**Results:**

Participants (intervention: *n* = 10; control: *n* = 9) recruited from 13 National Health Service hospital sites in the UK participated in the interviews. Two main themes, with sub-themes, were elicited from the data: mechanisms determining glove use: ‘As soon as your joints get a bit warmer, the pain actually eases’ (thermal qualities; glove use in daily activities; glove use during sleep); and ambivalence about benefits of arthritis gloves: ‘I suppose a normal pair of gloves would do the same sort of thing?’ (are they a help or hindrance?; aesthetic appeal; future use of gloves).

**Conclusion:**

Participants had ambivalent views on the impact of both the intervention and the loose-fitting placebo gloves on their hand pain and function, identifying warmth as the main benefit. Ordinary mid-finger-length gloves widely accessible from high street suppliers could deliver warmth and provide the perceived benefits to hand pain and function.

**Trial registration:** ISRCTN, ISRCTN25892131; registered 5 September 2016 : retrospectively registered.

Key Messages

- People with RA and undifferentiated inflammatory arthritis had ambivalent views about the impact of wearing both the Isotoner and the loose-fitting placebo gloves on their hand pain and function.
- Participants focused on the thermal qualities of the gloves, perceiving the provision of warmth as the main benefit.
- Ordinary, widely accessible mid-finger-length gloves providing warmth might provide similar benefits to therapist-prescribed arthritis gloves.

## Introduction

Hand pain and functional problems are common and a leading cause of disabilities in everyday activities, leisure and work in people with RA [1]. Nine out of 10 adults with RA report pain, stiffness, muscle weakness, paraesthesia and difficulty making a fist [2]. These symptoms can persist and deteriorate even when disease activity is controlled with DMARDs [3, 4], which are prescribed to achieve remission or lower disease activity and prevent radiographic progression of the disease. DMARDs are also prescribed to those with persistent synovitis who have not yet met the diagnostic criteria for RA [i.e. undifferentiated inflammatory arthritis (UIA)] [5].

Pain from RA is historically thought to be a direct result of peripheral inflammation [6], but later studies have shown discordance between clinicians’ assessments of inflammation and patient-reported pain [7, 8]. Knowledge and understanding of pain mechanisms in RA have since developed into describing this process as an interplay between joint pathology and the processing of pain signals by peripheral, spinal and supraspinal pain pathways [9]. Peripheral pain mechanisms include the direct activation of nociceptors, in addition to sensitization of nociceptors by joint inflammation [10, 11]. Like peripheral sensitization, central causes of pain arise as a result of abnormalities in the CNS and dysregulation of the CNS pain pathways, leading to chronic pain [12, 13]. These mechanisms might explain, in part, why a significant proportion of patients with RA remain symptomatic even with biological and targeted synthetic DMARDs (b/tsDMARDs) [13].

Arthritis gloves are widely prescribed in rheumatology departments by occupational therapists to people with RA and UIA presenting with hand pain and problems: for daytime wear to reduce hand pain and improve hand function and/or for night-time wear to reduce pain, improve sleep and reduce morning stiffness [14, 15]. The mechanism by which arthritis gloves impact on hand symptoms is thought to be through compression, which removes extracellular fluid (e.g. swelling), thus reducing pain and stiffness and improving finger movement [16, 17]. Different makes of glove apply differing amounts of pressure. Systematic review evidence was inconclusive about the effectiveness of arthritis gloves [15].

**Fig. 1:**
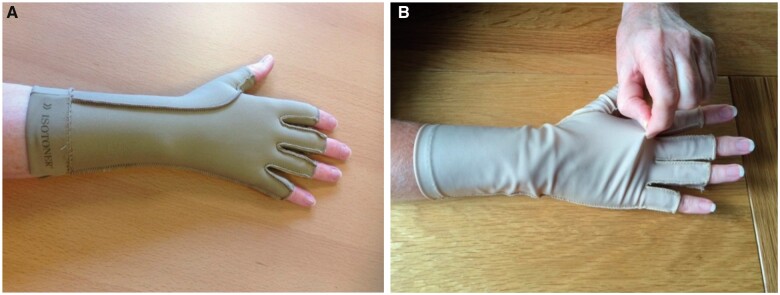
Arthritis gloves (intervention and control) (**A**) Intervention (Isotoner) arthritis glove. (**B**) Placebo glove (loose-fitting Jobskin classic oedema glove: control group).

We have previously reported results from a randomized controlled trial (RCT) in adults (≥18 years of age) with RA or UIA in the UK, investigating the clinical and cost-effectiveness of arthritis gloves compared with placebo gloves on hand pain, stiffness and function [1, 18]. Participants in the intervention group received correctly fitted three-quarter-length finger Isotoner gloves [19]. Participants in the control group received loose-fitting three-quarter-length finger Jobskin classic oedema gloves [20]. The placebo gloves for the control group were chosen by a panel of experts including occupational therapists, researchers and patient research partners to ensure their credibility. In the trial, they were fitted at least one size too large and exerted no or minimal pressure to ensure they did not apply therapeutic levels of compression [Fig. 1]. When fitting gloves, occupational therapists measured participants’ MCP joint circumference to determine the glove size required and used their clinical judgement to determine appropriate fit, following the *A-GLOVES Occupational Therapy Glove Provision Manual* [1, 21]. Therapists attended theoretical and practical training in intervention and placebo glove fitting, in order to standardize treatment delivery [22]. All participants received the same verbal and written information about glove wear and care, with the glove wear regimen individualized to suit their needs (i.e. wearing gloves in the day, night or both). All received written information about hand self-management (joint protection and exercise). Occupational therapists reviewed glove fit 2–4 weeks later or asked participants to contact them if experiencing problems. Given that gloves are intended to be worn long term, a 12-week follow-up was selected to allow several weeks for glove tolerance to develop and for participants to experience the effects of regular wear for ≤2 months across a range of activities [18].

This nested qualitative study aimed to explore participants’ views on the impact of wearing arthritis gloves on their hand pain and function to provide an insight into their lived experience and to gain a greater understanding of the contextual and person-related factors affecting arthritis glove wearing.

## Methods

### Study design and ethics

A qualitative design, nested within a RCT comparing National Health Service (NHS) Isotoner arthritis gloves with a loosely fitted placebo glove in people with RA and UIA, was used. The study aimed to explore narratives of participants’ views and experiences of glove wear. Data were collected using one-to-one, semi-structured, face-to-face and telephone interviews. The semi-structured interview schedule was developed by the research team, composed of occupational therapists, a rheumatology consultant, a nurse, patient research partners and qualitative methodologists (see Table 1).

**Table 1:** Core interview questions

Semi-structured interview schedule
Opening question: having worn the arthritis gloves for up to 12 weeks, could you tell me about any negative or positive effects these have had on your hand pain and hand problems?
Prompts: How did you find wearing them? How was it to put them on and off your hands? Were there any particular activities you found they helped with? Were there any particular activities you found they did not help with? Was there anything about the gloves or their effects which you think helped/hindered your hand pain and hand problems? Have you used them at work (if employed)? Did you have any problems wearing the gloves? What did you think of the gloves’ appearance? What did you think of the quality of the gloves you were given? How did you find cleaning them? Would you consider buying them in the future? Would you change anything about them to make it better for your use?

This study complies with the Declaration of Helsinki. The study was approved by the North of Scotland National Research Ethics Committee (REC reference 15/NS/0077) and Universities of Salford and Central Lancashire (Lancashire CTU). The study protocol and RCT results have been published [1, 18].

### Sampling and recruitment

The trial was conducted in rheumatology occupational therapy departments across 16 NHS sites in England and Scotland. Participants who gave written informed consent to take part in the optional qualitative interviews were sampled from diverse hospital sites in England and Scotland using maximum variation purposive sampling [23] to capture a wide range of perspectives. Participants in each group were selected by the Lancashire Clinical Trials Unit progressively throughout the study to include a male-to-female ratio representative of the study population and a range of ages, baseline hand pain (mild, moderate or severe) and 12-week self-reported levels of adherence with glove wear. Potential participants were contacted by telephone following completion of the 12-week follow-up questionnaire to check whether they were still willing to be interviewed. If so, they were given the option to participate in the interview either by telephone or face to face, at a mutually convenient date and location.

### Data collection

The interviewer and participants were blinded to the treatment allocation and had no prior acquaintance. Before the start of the interviews, participants’ demographic data (age, sex, disease duration and medication) were obtained with their informed consent [24]. Interviews were audio-recorded, transcribed verbatim by a professional transcription service, checked for accuracy against the original audio files, and anonymized with pseudonyms and replacement words for people and places to maintain confidentiality and allow data sharing. Participants were given the chance to review their interview transcript.

### Analysis

An inductive thematic analysis was conducted [25]. This approach was taken to examine different perspectives, highlighting similarities and differences and generating rich insights from the data [25]. The lead author read and coded all 19 transcriptions using NVivo 12 software [26]. Related clusters of coded text were then grouped together conceptually to form sub-themes under the main themes to describe the interpretation of the data. A sub-set of 10 anonymized transcripts were then analysed independently by two other members of the study team to confirm the themes arising from the data [27]. The group allocation of the participants was revealed after the initial coding of the data by the study team to aid the interpretation of themes and allow comparisons across the views and experiences of participants from the intervention and control groups. The final analysis reported is based on the combined interpretation of the data by the study team.

## Results

Nineteen participants were interviewed (10 intervention and 9 control). They ranged in age from 30 to 79 years; 12 were women; 17 had a diagnosis of RA and two UIA; time since diagnosis ranged from 4 months to 22 years; four were employed, one unemployed, five identified as homemakers, and nine were retired (two of whom had retired early owing to ill health) (Table 2).

**Table 2:** Demographic and clinical data

Number	Sex	Age (years)	Diagnosis	TSD (months)	Medication regimen	**TG** ^e^
1	M	57	RA	11	1 DMARD	INT
2	M	79	UIA	4	≥2 DMARDs	INT
3	M	75	RA	11	1 DMARD	INT
4	F	69	RA	3	1 DMARD + opioids (PRN)	CNT
5	F	67	RA	240	1 DMARD + opioids (PRN)	INT
6	M	49	RA	84	1 DMARD	INT
7	F	54	RA	60	1 DMARD	CNT
8	F	58	RA	12	NSAIDs + paracetamol	INT
9	F	73	RA	264	Biologics + paracetamol	INT
10	M	71	RA	48	1 DMARD	INT
11	F	61	RA	252	≥2 DMARDs + NSAIDs	CNT
12	F	59	RA	44	Biologics	INT
13	F	60	RA	120	≥2 DMARDs	CNT
14	F	52	RA	216	Biologics	CNT
15	F	50	UIA	228	≥2 DMARDs	CNT
16	F	30	RA	24	≥2 DMARDs	CNT
17	M	63	RA	216	Biologics + NSAIDs	INT
18	M	70	RA	12	NSAIDs	CNT
19	F	74	RA	10	≥2 DMARDs + amitriptyline^a^	CNT

a Amitriptyline: a tricyclic antidepressant used for pain management.

CNT: control/placebo glove; F: female; INT: intervention glove; M: male; PRN: When required; TG: treatment group; TSD: time since diagnosis (at baseline); UIA: undifferentiated inflammatory arthritis.

Two main themes emerged, with three sub-themes under each theme (Table 3). The themes and interpretations of sub-themes are detailed below. Data excerpts to evidence the themes and sub-themes are provided in Tables 4 and 5.

**Table 3:** Labels of main and sub-themes

Main theme	Sub-themes
1. Mechanisms determining glove use	Thermal qualities
	Glove use in daily activities
	Glove use during sleep
2. Ambivalence about the benefit of the gloves	Are they a help or hindrance?
	Aesthetic appeal
	Durability and maintenance of gloves

**Table 4:** Data excerpts evidencing theme 1 and sub-themes

Theme 1: mechanisms determining glove use: ‘As soon as your joints get a bit warmer, the pain actually eases’
(i) Thermal qualities
I think it was the support or the heat; I don’t know. And as I say, I tried to analyse it and I just couldn’t because really when you look at them you think it shouldn’t help you, but they do’. (F, 59 years old, CNT)
I found them very pleasant, it was quite elastic, warm, and I think warmth makes a big difference with the rheumatics in the hands. And in its own way comforting, but unfortunately it doesn’t seem to have helped me a lot. (M, 79 years old, INT)
They were fab. They actually made my joints warm and … as soon as your joints get a bit warmer, the pain actually eases. (M, 63 years old, CNT)
The only thing that I do like about them was they kept your hands warm in the cold. (F, 61 years old, CNT)
I didn’t use them when I went to bed at night … I found them very warm. (F, 69 years old, INT)
Well, especially in this weather, this cold weather … warm … they keep my wrists warm and that does help ’cause when it is colder they do go sore then, but they do help when it’s cold weather. (M, 75 years old, INT)
(ii) Glove use in daily activities
It helped a great deal with support, when I was doing housework … carrying shopping bags. It helped then. (M, 71 years old, INT)
…like, for instance, if I was to change a plug … or plug a device in, like headphones or something, I could actually do it quite simply because my fingers were … my hands weren’t cold … plug it into the device or whatever with the dexterity. (M, 63 years old, CNT)
they are a comfort … especially when I’m reading and yes, it’s great. (F, 73 years old, INT)
I don’t put them on when I do big jobs or anything like that … you know, vacuuming up and things like that … general household work. I don’t put them on when I’m washing up ’cause they’d get wet of course. (M, 75 years old, INT)
…because of course gloves, you can’t wear it if I’m driving because they’re a bit slippery and it’s not good for driving. (F, 30 years old, INT)
I could wear them all day, but when I try and do some cleaning or whatever, I have to take them off and then I’m like I can’t get these wet. (M, 70 years old, CNT)
…so I didn’t wear it if I was doing anything, like washing dishes or things like that, hoovering, I’d take them off. (F, 61 years old, CNT)
Well yes, anything where I had to grip things particularly, like the steering wheel for driving, or … I was very careful lifting crockery and such because I thought they were a little bit slippery. (F, 67 years old, CNT)
Social activities, outside, no. But as I say, it was either lifting a glass of beer or dealing cards and not helpful in either case. (M, 79 years old, INT)
(iii) Glove use during sleep
I mostly wear it at night because … my mornings are worse. So, if I wear it at night this helps me in the morning. You know, my wrist, it won’t get stuck. (F, 30 years old, INT)
I find when I wake up in the morning, they seem a lot easier, still stiff and still a bit swollen, but they’re better since I’ve been wearing the gloves. (M, 70 years old, CNT)
…at night-time I put them on when I go to bed. Not every night, it depends how my wrists feel, but they’re really comfortable … you don’t know you’ve got them on. (M, 79 years old, INT)
It was just, I think overnight with the amount of time that I was wearing them, clearly it didn’t agree with my system. (F, 58 years old, INT)

CNT: control group; F: female; INT: intervention group; M: male.

**Table 5:** Data excerpts evidencing theme 2 and sub-themes

Theme 2: ambivalence about the benefit of the gloves: ‘I suppose a normal pair of gloves would do the same sort of thing?’
(i) Are they a help or hindrance?
I was, like I say, taking them off and on, like … the instructions say keep on taking them off and then putting them on. And then I’d think, oh, forget this, I can’t be bothered taking them on and off, so I’d leave them off. (M, 70 years old, CNT)
I can’t say that I found them particularly helpful, as I say, apart from the comfort factor of having the warmth on my hands … but I think that’s probably the only benefit, I think. (F, 67 years old, CNT)
You get a slight warming in the hands, which is obviously beneficial. Apart from that, I can’t think of anything really. (M, 57 years old, INT)
…my hands sort of just go, oh, I’m pleased they are on. It seems to give them a little bit of a hand, if that makes sense. (M, 49 years old, CNT)
Yes, having the glove on seemed to keep my hands quite … so it helped my hands to move easier, particularly in the colder weather. (F, 67 years old, CNT)
I don’t like them. I felt it affected my hands more in them. I felt my hand was sore quite a lot. (F, 61 years old, CNT)
In the early days it was very helpful, but as time went by my pain in my hands got less. Whether that was the gloves, the exercise or the medication I’m not sure. (M, 79 years old, INT)
I went into it with a positive attitude, hoping that this would be something that would perhaps be an alternative to medication or at least, you know, the possibility that I would be able to take less medication, but unfortunately it didn’t work for me. (F, 58 years old, INT)
(ii) Aesthetic appeal
I think they are really nice, and they are like, I can hide my hands with them, that’s what I like. (F, 73 years old, INT)
…They felt alright. They looked alright. I don’t think anybody … nobody has sort of made any snide comments about them at all. (M, 70 years old, CNT)
I thought they actually looked, you know, quite attractive … lot of women, more so possible than men, feel that they want to hide their hands a little bit, and the fact that they were flesh coloured, I think they looked quite modern. And unobtrusive as well. (F, 58 years old, INT)
I think, because I’m fairly vain, and I just didn’t want to wear that colour, people do tend to, especially if you’re in the supermarket or doing anything, your hands are being used all the time, aren’t they, and they just sort of look at you as if to say, well, what are you hiding under there, you know, is it something I could catch? (F, 59 years old, CNT)
I mean, people will notice, but I’m sure they noticed my hands a lot more because they are quite ugly. (F, 73 years old, INT)
The fact that you’re conscious that, you know, you’ve got them on and if you’re, say, going shopping with them on or something like that, if people, you know, see them and they’re looking at you … I think I’d have had to take them off if I was going out. (F, 74 years old, CNT)
(iii) Future use of gloves
…it’s not a thing I’d go out and think, oh right, I’d better go to town and get a pair of them. No. Or go and buy them in the hospital or whatever, no. I can’t see any point in that … I suppose a normal pair of gloves would do the same sort of thing. (M, 70 years old, CNT)
I must admit I did find that the stitching around the cuffs of the wrist left a little bit to be desired. The stitching on mine has come undone. (M, 71 years old, INT)
Well in fact, if I had that same … the right conditions, and the hospital offered me to try them again, I’d probably give them a go, but as it is now I wouldn’t go out and buy any. (F, 50 years old, CNT)

CNT: control group; F: female; INT: intervention group; M: male.

### Theme 1: mechanisms determining glove use: ‘As soon as your joints get a bit warmer, the pain actually eases.’

Participants in both groups mainly used the gloves indoors to keep hands warm during rest and while carrying out light domestic activities, outdoors to aid activities such as carrying shopping bags and doing light gardening, and at night to help with sleep and morning stiffness. Gloves were not used for activities that would involve the gloves getting wet or dirty or that would be unsafe while wearing the gloves, owing to the slippery nature of the gloves making it hard to grip objects. Participants also avoided wearing gloves when they were socializing or shopping, because taking the gloves off and putting them back on again was found to be bothersome. Some patients found wearing the gloves during sleep comfortable and helpful, whereas others reported adverse reactions (see Table 4).

#### Thermal qualities

Participants commented on the thermal qualities of the Isotoner and placebo gloves, suggesting that the warmth and comfort they provided helped to ease their joints and relieve stiffness. Most participants attributed the therapeutic effect of the gloves to the feeling of warmth, and they explained that cold weather made their symptoms worse. They believed that the warmth helped their hand pain and function, because their joints felt ‘looser’. Some participants did not think that the gloves helped at all, despite their warming effect.

#### Glove use in daily activities

Gloves were mainly used during sedentary daily activities, such as relaxing in front of the television, reading a book or doing light housework that did not require a firm grip (e.g. tidying) or getting their hands wet. Participants considered that donning and doffing the gloves were difficult and therefore refrained from wearing them when they were doing activities that required them to do this frequently (i.e. needing a firm grip or getting hands wet). Most people handwashed the gloves to avoid them fraying at the edges, which meant they could not easily wear the same gloves day and night because they needed time to dry flat. Owing to the slippery nature of the gloves, operating machinery and driving were deemed dangerous, and gloves were not worn during these activities. Although most participants were happy to wear them outdoors, like normal gloves to keep their hands warm and to carry shopping bags, they were not worn during socializing or shopping, because the participants were either self-conscious about wearing them, or the gloves were deemed unhelpful when using their hands (e.g. taking coins out of a purse or lifting a cup).

#### Glove use during sleep

Participants who wore the gloves at night to help with night pain and morning stiffness found them helpful because of the warming and comforting effect on their hands and wrists. They reported that their hands were still a bit swollen and stiff in the mornings, but that it was easier to ‘get going’. Some participants did not like the feeling of going to bed with either Isotoner or placebo gloves, reporting that they felt restrictive and uncomfortable. Others added that they stopped wearing them at night because they felt that wearing them for long, uninterrupted hours made their hands sore and increased swelling (for both types of gloves).

### Theme 2: ambivalence about the benefit of the gloves: ‘I suppose a normal pair of gloves would do the same sort of thing?’

Participants were generally ambivalent about the benefit of wearing gloves on their hand pain and function. Some could not tell if their hand pain or function got better or worse. Others found the gloves more of a hindrance then a help, owing to having to take them on and off during activities that required getting their hands wet or required a firm grip. Those who found the gloves beneficial attributed this solely to the warmth and comfort they felt when wearing the gloves and considered that the therapeutic effects lasted only while wearing the gloves, because their symptoms often returned. The gloves were not seen as a medical device or an orthotic prescription, despite being fitted by occupational therapist in NHS clinics. Instead, they were perceived as ordinary gloves. Some participants were disappointed, because they had not perceived any benefits from wearing them.

#### Are they a help or hindrance?

Participants were ambivalent regarding the help *vs* hindrance of the gloves in daily life and how appropriate it would be to wear the gloves during warm temperatures. They said their hand pain and condition changed from day to day and were not sure whether any benefits perceived were attributable to fluctuating symptoms or other factors (e.g. changes in the weather or in the dose of pain medication). Most people could not think of any other benefits from the gloves other than providing warmth. Some people did not like the gloves at all and found that wearing them made their hands sore and painful because overheating. This was true for both the intervention and control gloves.

#### Aesthetic appeal

Some participants found the appearance of the gloves acceptable and unobtrusive and liked the fact that they could cover what they perceived to be their embarrassing hand appearance, whereas others had strong opinions on wearing the gloves when socializing, suggesting that they would not want to ‘announce’ that something was wrong with their hands.

#### Future use of gloves

Most people who used the gloves during the daytime complained about the durability of the gloves and the need to repair/replace the gloves often, because they frayed or ripped around the stitches after only a few weeks of wear. Those who used the gloves only at night to help with sleep found that this was less of a problem, but the consensus was that they would not go out in a rush to purchase them if they had to pay for the gloves themselves. Some participants said that if the hospital offered them another pair to try, they might consider trying them again, but others suggested that they would not want to use them again, even if they were recommended and provided by their hospital for free.

## Discussion

This study aimed to explore the role of person-related and contextual factors on arthritis glove wearing to expand on the findings of the RCT by considering patients’ lived experience. Our findings indicated that, although most welcomed the feeling of warmth the gloves provided, the impact of this on their hand pain was limited, and gloves were often reported as not contributing to improvement in hand functional ability. It was implicit within the themes that participants had similar ambivalent views about the impact of wearing the closely fitted intervention and the loosely fitted placebo/control gloves, with no distinctions in how they were used, the perceived impact, difficulties experienced or aesthetic appeal.

Before this study, no qualitative studies had explored the contextual effects of wearing arthritis gloves from the patients’ perspective. In comparison to qualitative studies that investigated the use and preference of wrist splints [28] and the determinants of wrist splint use [29] from RA patients’ perspectives, similar themes were reported for arthritis glove use [28, 29]. Wrist splints are provided to people with RA who are struggling with hand pain and function (e.g. grip strength and manual dexterity) to protect the wrists during daily activities that might overstrain the joints, which can help with pain and reduce the signs of inflammation [30]. The reasons for wearing and not wearing wrist working splints were related to intentional decisions of the participants, which were based primarily on perceived benefits and barriers of splint wearing. The views about what was perceived as a benefit or a barrier varied greatly among the participants interviewed [29]. Many reported wrist support and rest/immobilization of the wrist as supplementary reasons for wearing the wrist splint, whereas others simultaneously reported these as disadvantages, because the splint restricted performance of activity. Likewise, some participants indicated improvement in functional ability owing to splint wear, whereas others experienced decreased functional ability [29]. In a similar way, in our study the participants were generally ambivalent about the benefit of wearing arthritis gloves, as they weighed the pros and cons in relationship to improvement on their hand pain and function *vs* the practical and social limitations caused by wearing the arthritis gloves. The major disadvantages of wrist splints were cited as the follows: having to take off the splint to perform many activities because it gets wet and dirty easily; its long drying time; unpleasant physical contact with the splint because of the hard metal stay; sweating; wear and tear; prohibited ability to drive a car; and inability to take off the splint independently [29]. Again, arthritis glove wearers reported similar issues in relationship to having to take them on and off during activities that required getting their hands wet or required a firm grip, having to hand wash the gloves frequently and the long drying time to allow re-usage. Some participants were neutral, whereas others were negative about the appearance of their splint. Those who were neutral thought its appearance was ‘not important’ to them, whereas those with negative views remarked on the material, straps, metal stay and/or side effects of the splint. Most importantly, for some patients, these complaints were reason enough to take off the splint [29]. This pattern was also apparent on the arthritis glove wearers, because some participants found the appearance of the gloves acceptable, whereas others perceived wearing these as ‘embarrassing’ and therefore refrained from wearing them when socializing.

In this qualitative study, although there were no differences reported in hand pain and function in participants who received the intervention or the placebo gloves, both groups reported some relief in hand symptoms attributable to the warmth gloves afforded. Although for a long time considered ineffective, placebo treatments now have an accepted role in research and clinical practice, because studies have shown their therapeutic value [31]. Placebo and nocebo (i.e. a situation where a negative outcome occurs owing to a belief that the intervention will cause harm) represent complex and distinct psychoneurobiological phenomena, in which behavioural and neurophysiological changes follow the application of a treatment [32]. The therapist and the patient’s characteristics, the patient–therapist relationship and the characteristics of the treatment are all contextual factors influencing clinical outcomes [32, 33]. Thus, when evaluating the effectiveness of treatments, the importance of patients’ expectations, feelings and clinical context should not be ignored [33]. The placebo effect might have played a role in participants’ appraisal of arthritis glove wearing, because both gloves were prescribed and fitted by rheumatology occupational therapists in a hospital setting, and the placebo/control gloves had the credible appearance of arthritis gloves. Nonetheless, many people with RA believe that weather and the seasons affect their symptoms, and the effect of temperature on RA pain has been reported previously [34–37], especially the effect of extreme temperatures [38]. Thus, it is unsurprising that participants identified the therapeutic effect of ‘warmth’ as the reason why the gloves might have helped their hand symptoms, because gloves could have compensated for the impact of cold temperatures, and this also provided comfort.

Participants were also ambivalent across the board about their future use of arthritis gloves, especially if they had to purchase the gloves themselves, because they had concerns around both the perceived benefits and the costs of the gloves to make this worthwhile. They likened the arthritis gloves to ‘normal’ ordinary outdoor gloves, other than their medical appearance and slippery nature, and some had concerns about wearing these when socializing. Ordinary light-weight three-quarter-finger gloves, made of nylon, cotton or wool (according to the person’s preference), purchased online or from a high street store, could lead to the same benefits at reduced cost.

### Strengths and limitations

The strengths of this study were that both the participants and the interviewer were blinded to group allocation, and the interviews were conducted shortly after 12 weeks of glove wearing to ensure that the experience was still fresh in the participants’ minds. Participants were representative of those referred to receive arthritis gloves, including those newly diagnosed and those with long-standing disease. The study team analysing the data had a variety of backgrounds, including patient partners with lived experience of glove wearing, academics and clinicians who work in rheumatology settings. A study limitation was that most interviews were conducted by telephone (*n* = 16) owing to geographical limitations, with only three interviews being held face to face. Different interview modes might yield different results, with face-to-face interviews having long been deemed as the superior interview technique in qualitative research [39, 40]. However, comparisons of the interview transcripts showed no significant differences in the quality of the data obtained between the two interview modes.

### Conclusion

Participants were ambivalent about the impact of the intervention and placebo gloves on their hand pain and function, identifying warmth as the most likely therapeutic benefit. There were no noteworthy differences in the appraisal of either of the two types of gloves for ease of wear, symptom management or appearance to suggest that one was perceived as better than the other. Within both groups there were differing views on whether the gloves were a help or a hindrance to their hand function and whether the participants would consider purchasing them in the future to manage their hand symptoms, although some were willing to try them again if prescribed by a therapist. Given that warmth was identified as the most beneficial therapeutic effect, most patients with arthritis could obtain the same benefits from ordinary mid-finger-length gloves, without the need for a clinical prescription for an arthritis glove from rheumatology services. Future research should investigate whether people with arthritis would consider wearing ordinary light-weight gloves to evaluate whether these have similar benefits.
